# Phenyl 2,3,4-tri-*O*-benzyl-1-thio-α-d-mannopyran­oside monohydrate

**DOI:** 10.1107/S1600536810019604

**Published:** 2010-06-05

**Authors:** Maxime Durka, Bernadette Norberg, Yvain Roué, Stéphane P. Vincent, Johan Wouters

**Affiliations:** a Department of Chemistry, University of Namur, 61 Rue de Bruxelles, B-5000 Namur, Belgium

## Abstract

In the title compound, C_33_H_34_O_5_S·H_2_O, the mannopyran­oside ring adopts a chair conformation with the 2-α-thio­phenyl group occupying an axial position. One of the pendant benzyl groups is disordered over two sets of sites in a 0.5:0.5 ratio. In the crystal, the water mol­ecule makes two O—H⋯O hydrogen bonds to an adjacent sugar mol­ecule with the O atoms of the primary alcohol and ether groups acting as acceptors. At the same time, the OH group of the sugar makes a hydrogen bond to a water mol­ecule.

## Related literature

For background to the synthesis and properties of mannopyran­osides, see: Boons (1991 ▶); Szurmai *et al.* (1994 ▶); Caravano *et al.* (2003 ▶); Grizot *et al.* (2006 ▶); Dohi *et al.* (2008 ▶). For ring conformation analysis, see: Cremer & Pople (1975 ▶).
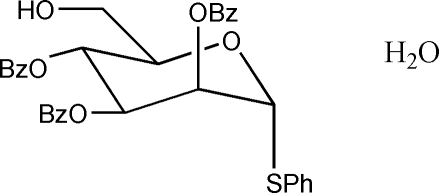


## Experimental

### 

#### Crystal data

C_33_H_34_O_5_S·H_2_O
*M* *_r_* = 560.69
Monoclinic, 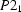

*a* = 12.628 (1) Å
*b* = 8.084 (1) Å
*c* = 14.832 (2) Å
β = 101.380 (5)°
*V* = 1484.4 (2) Å^3^
*Z* = 2
Mo *K*α radiation
μ = 0.15 mm^−1^
*T* = 150 K
0.35 × 0.30 × 0.17 mm

#### Data collection

Oxford Diffraction Gemini Ruby CCD diffractometer
Absorption correction: multi-scan (*CrysAlis PRO*; Oxford Diffraction, 2009 ▶) *T*_min_ = 0.948, *T*_max_ = 0.974
12650 measured reflections
5205 independent reflections
4333 reflections with *I* > 2σ(*I*)
*R*_int_ = 0.022

#### Refinement

*R*[*F* ^2^ > 2σ(*F* ^2^)] = 0.030
*wR*(*F* ^2^) = 0.062
*S* = 0.97
5205 reflections
400 parameters
17 restraints
H atoms treated by a mixture of independent and constrained refinement
Δρ_max_ = 0.13 e Å^−3^
Δρ_min_ = −0.17 e Å^−3^
Absolute structure: Flack (1983 ▶), 2381 Friedel pairs
Flack parameter: 0.01 (5)

### 

Data collection: *CrysAlis CCD* (Oxford Diffraction, 2009 ▶); cell refinement: *CrysAlis RED* (Oxford Diffraction, 2009 ▶); data reduction: *CrysAlis RED*; program(s) used to solve structure: *SHELXS97* (Sheldrick, 2008 ▶); program(s) used to refine structure: *SHELXL97* (Sheldrick, 2008 ▶); molecular graphics: *PLATON* (Spek, 2009 ▶); software used to prepare material for publication: *PLATON*.

## Supplementary Material

Crystal structure: contains datablocks I, global. DOI: 10.1107/S1600536810019604/hb5424sup1.cif

Structure factors: contains datablocks I. DOI: 10.1107/S1600536810019604/hb5424Isup2.hkl

Additional supplementary materials:  crystallographic information; 3D view; checkCIF report

## Figures and Tables

**Table 1:** Hydrogen-bond geometry (Å, °)

*D*—H⋯*A*	*D*—H	H⋯*A*	*D*⋯*A*	*D*—H⋯*A*
O*W*—H*W*1⋯O2	0.88 (3)	2.00 (3)	2.861 (2)	166 (2)
O*W*—H*W*2⋯O6	0.78 (2)	2.07 (2)	2.827 (2)	165 (2)
O6—H6⋯O*W*^i^	0.87 (2)	1.89 (2)	2.745 (2)	169 (2)

Symmetry code: (i) 
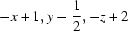
.

**Fig. 1.**
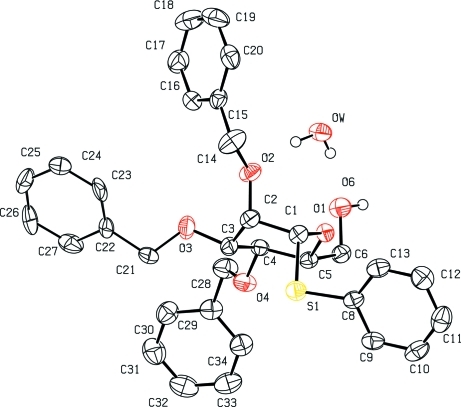
The molecular structure of (I): C-bound H atoms are omitted and disorder is not presented for clarity. Displacement ellipsoids for non-H atoms are drawn at the 50% probability level.

## supplementary crystallographic information

### Comment

Oligosaccharides are natural ligands of lectins, which are important receptors involved in key biological processes. Efficient strategies for the regioselective transformations of monosaccharides to achieve glycosylation reactions or further functionalizations are of paramount importance. Furthermore, the knowledge of the conformations of carbohydrates alone or bound to protein gives access to key informations that can be exploited to understand biocatalytic processes or stereoelectronic effects (Caravano *et al.*, 2003).

Numerous strategies of protections of mannopyranosides have been described in the litterature (*e.g.* Boons, 1991; Szurmai *et al.*, 1994) in order to derivatise the primary alcohol. The title compound is also a key intermediate for the synthesis of heptosyl transferase inhibitors (Dohi *et al.*, 2008; Grizot *et al.*, 2006).

Crystal structure of the title compound confirms the expected relative stereochemistry : C1 *R*, C2 *S*, C3 *S*, C4 *R*, C5 *R*. The mannopyranoside adopts a chair conformation with puckering amplitude (Q) = 0.522 (3) Å, Theta = 3.6 (3) °, and Phi = 60 (5) ° (Cremer & Pople, 1975).

The 2-alpha-thiophenyl group on C1 and the *O*-benzyl group on C2 occupy an axial position, the two other *O*-benzyl groups (on C3 and C4) and carbon atom C6 occupying an equatorial position.

Thiophenyl-2,3,4-*O*-tri-benzyl-alpha-*D*-mannopyranoside co-crystallized with one water molecule (O_W_). This water molecule is part of a H bond network involving the primary alcohol O6 and also secondary alcohol O2 (Table 1). Packing is further reinforced by van der Waals interactions involving the aromatic rings.

### Experimental

The title compound was obtained by a six-step synthetic route that will be described in details elsewhere. Overall yield is 70%. Colourless prisms of (I) were obtained by evaporation of a solution in ethyl acetate.

### Refinement

Disorder of the benzyl C(21) > C(27) moiety substituting oxygen O(4) was included in the refinement (0.5 occupacy for both parts that were restrained to have similar bond lengths and angles). H atoms on water oxygen atom O_W_ and on alcohol oxygen atom O(6) were located by Fourier difference maps and allowed to ride on their parent O atoms.

All other H atoms were placed at idealized positions and allowed to ride on their parent atoms, with C—H = 0.97 Å and U_iso_(H) = 1.2U_eq_(C) for methylene groups, C—H = 0.93 Å and U_iso_(H) = 1.2U_eq_(C) for aromatic carbons, and C—H = 0.96 Å and U_iso_(H) = 1.5U_eq_(C) for the methyl group.

### Crystal data

**Table:**

C_33_H_34_O_5_S·H_2_O	*F*(000) = 596.0
*M**_r_* = 560.69	*D*_x_ = 1.255 Mg m^−^^3^
Monoclinic, *P*2_1_	Mo *K*α radiation, λ = 0.71073 Å
Hall symbol: P 2yb	Cell parameters from 7534 reflections
*a* = 12.628 (1) Å	θ = 3.0–28.2°
*b* = 8.084 (1) Å	µ = 0.15 mm^−^^1^
*c* = 14.832 (2) Å	*T* = 150 K
β = 101.380 (5)°	Prism, colourless
*V* = 1484.4 (2) Å^3^	0.35 × 0.30 × 0.17 mm
*Z* = 2	

### Data collection

**Table:**

Oxford Diffraction Gemini Ruby CCD diffractometer	5205 independent reflections
Radiation source: Enhanced fine-focus sealed tube	4333 reflections with *I* > 2σ(*I*)
graphite	*R*_int_ = 0.022
ω scans	θ_max_ = 25.0°, θ_min_ = 3.0°
Absorption correction: multi-scan (*CrysAlis PRO*; Oxford Diffraction, 2009)	*h* = −15→14
*T*_min_ = 0.948, *T*_max_ = 0.974	*k* = −9→9
12650 measured reflections	*l* = −17→17

### Refinement

**Table:**

Refinement on *F*^2^	Secondary atom site location: difference Fourier map
Least-squares matrix: full	Hydrogen site location: inferred from neighbouring sites
*R*[*F*^2^ > 2σ(*F*^2^)] = 0.030	H atoms treated by a mixture of independent and constrained refinement
*wR*(*F*^2^) = 0.062	*w* = 1/[σ^2^(*F*_o_^2^) + (0.0359*P*)^2^] where *P* = (*F*_o_^2^ + 2*F*_c_^2^)/3
*S* = 0.97	(Δ/σ)_max_ = 0.001
5205 reflections	Δρ_max_ = 0.13 e Å^−^^3^
400 parameters	Δρ_min_ = −0.17 e Å^−^^3^
17 restraints	Absolute structure: Flack (1983), 2381 Friedel pairs
Primary atom site location: structure-invariant direct methods	Flack parameter: 0.01 (5)

### Special details

**Table:**

Geometry. All esds (except the esd in the dihedral angle between two l.s. planes) are estimated using the full covariance matrix. The cell esds are taken into account individually in the estimation of esds in distances, angles and torsion angles; correlations between esds in cell parameters are only used when they are defined by crystal symmetry. An approximate (isotropic) treatment of cell esds is used for estimating esds involving l.s. planes.
Refinement. Refinement of *F*^2^ against ALL reflections. The weighted *R*-factor wR and goodness of fit *S* are based on *F*^2^, conventional *R*-factors *R* are based on *F*, with *F* set to zero for negative *F*^2^. The threshold expression of *F*^2^ > σ(*F*^2^) is used only for calculating *R*-factors(gt) etc. and is not relevant to the choice of reflections for refinement. *R*-factors based on *F*^2^ are statistically about twice as large as those based on *F*, and *R*- factors based on ALL data will be even larger.

### Fractional atomic coordinates and isotropic or equivalent isotropic displacement parameters (Å^2^)

**Table:**

	*x*	*y*	*z*	*U*_iso_*/*U*_eq_	Occ. (<1)
C1	0.28677 (14)	0.7097 (2)	0.89516 (11)	0.0300 (4)	
H1	0.3281	0.7834	0.9416	0.036*	
C2	0.32672 (13)	0.7370 (2)	0.80557 (11)	0.0334 (4)	
H2	0.3072	0.8487	0.7825	0.040*	
C3	0.27664 (14)	0.6105 (2)	0.73342 (11)	0.0337 (4)	
H3	0.1995	0.6349	0.7142	0.040*	
C4	0.28925 (14)	0.4357 (2)	0.77082 (10)	0.0297 (4)	
H4	0.3641	0.3989	0.7759	0.036*	
C5	0.25565 (14)	0.4210 (2)	0.86404 (10)	0.0276 (4)	
H5	0.1771	0.4349	0.8547	0.033*	
C6	0.28498 (13)	0.2565 (2)	0.90995 (11)	0.0323 (4)	
H6A	0.2635	0.2547	0.9692	0.039*	
H6B	0.2466	0.1688	0.8725	0.039*	
C8	0.11808 (13)	0.7620 (2)	0.98996 (11)	0.0308 (4)	
C9	0.02534 (15)	0.6816 (2)	1.00193 (13)	0.0370 (5)	
H9	−0.0181	0.6284	0.9525	0.044*	
C10	−0.00307 (16)	0.6802 (2)	1.08781 (14)	0.0448 (5)	
H10	−0.0665	0.6283	1.0954	0.054*	
C11	0.06208 (17)	0.7552 (3)	1.16149 (13)	0.0489 (5)	
H11	0.0431	0.7537	1.2190	0.059*	
C12	0.15545 (18)	0.8325 (3)	1.14995 (13)	0.0453 (5)	
H12	0.2004	0.8810	1.2002	0.054*	
C13	0.18316 (15)	0.8388 (2)	1.06442 (13)	0.0378 (5)	
H13	0.2453	0.8945	1.0568	0.045*	
C14	0.50097 (18)	0.8655 (2)	0.81855 (16)	0.0507 (6)	
H14A	0.5083	0.9246	0.8764	0.061*	
H14B	0.4635	0.9367	0.7699	0.061*	
C15	0.61013 (15)	0.8228 (2)	0.80096 (11)	0.0322 (4)	
C16	0.62073 (15)	0.7141 (2)	0.73104 (11)	0.0367 (4)	
H16	0.5595	0.6650	0.6961	0.044*	
C17	0.72045 (17)	0.6782 (3)	0.71287 (13)	0.0478 (5)	
H17	0.7264	0.6048	0.6659	0.057*	
C18	0.81128 (17)	0.7497 (3)	0.76340 (17)	0.0603 (6)	
H18	0.8787	0.7253	0.7505	0.072*	
C19	0.80278 (18)	0.8570 (3)	0.83291 (16)	0.0578 (7)	
H19	0.8647	0.9052	0.8673	0.069*	
C20	0.70225 (17)	0.8946 (2)	0.85263 (12)	0.0434 (5)	
H20	0.6968	0.9673	0.9001	0.052*	
O3	0.32582 (10)	0.61829 (18)	0.65476 (8)	0.0464 (4)	
C21	0.2578 (3)	0.6608 (6)	0.5714 (2)	0.0441 (11)	0.50
H21A	0.2135	0.7550	0.5805	0.053*	0.50
H21B	0.2102	0.5689	0.5495	0.053*	0.50
C22	0.3247 (5)	0.7026 (7)	0.5018 (4)	0.0280 (18)	0.50
C23	0.4047 (6)	0.8186 (9)	0.5201 (3)	0.043 (2)	0.50
H23	0.4207	0.8676	0.5779	0.051*	0.50
C24	0.4617 (5)	0.8638 (7)	0.4547 (5)	0.0407 (15)	0.50
H24	0.5178	0.9396	0.4692	0.049*	0.50
C25	0.4364 (5)	0.7974 (10)	0.3673 (5)	0.0456 (16)	0.50
H25	0.4748	0.8301	0.3229	0.055*	0.50
C26	0.3545 (7)	0.6830 (9)	0.3456 (3)	0.0481 (19)	0.50
H26	0.3372	0.6388	0.2866	0.058*	0.50
C27	0.2978 (6)	0.6338 (10)	0.4127 (4)	0.044 (2)	0.50
H27	0.2427	0.5562	0.3988	0.053*	0.50
C21'	0.2980 (4)	0.7526 (6)	0.5997 (2)	0.0413 (7)	0.50
H21C	0.3253	0.8515	0.6334	0.050*	0.50
H21D	0.2199	0.7608	0.5844	0.050*	0.50
C22'	0.3411 (10)	0.7456 (13)	0.5118 (7)	0.0413 (7)	0.50
C23'	0.4273 (9)	0.8369 (12)	0.4994 (6)	0.0413 (7)	0.50
H23'	0.4617	0.9043	0.5471	0.050*	0.50
C24'	0.4653 (6)	0.8333 (8)	0.4192 (7)	0.0413 (7)	0.50
H24'	0.5225	0.9015	0.4122	0.050*	0.50
C25'	0.4206 (7)	0.7315 (9)	0.3503 (6)	0.0413 (7)	0.50
H25'	0.4465	0.7287	0.2958	0.050*	0.50
C26'	0.3361 (8)	0.6316 (8)	0.3617 (6)	0.0413 (7)	0.50
H26'	0.3052	0.5596	0.3149	0.050*	0.50
C27'	0.2970 (8)	0.6377 (14)	0.4420 (8)	0.0413 (7)	0.50
H27'	0.2405	0.5687	0.4494	0.050*	0.50
C28	0.26726 (15)	0.2153 (3)	0.66005 (13)	0.0451 (5)	
H28A	0.3146	0.1422	0.7016	0.054*	
H28B	0.3101	0.2731	0.6224	0.054*	
C29	0.17992 (15)	0.1171 (2)	0.60016 (12)	0.0376 (5)	
C30	0.17758 (17)	0.1035 (3)	0.50676 (13)	0.0445 (5)	
H30	0.2310	0.1538	0.4812	0.053*	
C31	0.09579 (19)	0.0151 (3)	0.45124 (14)	0.0540 (6)	
H31	0.0945	0.0064	0.3885	0.065*	
C32	0.01714 (19)	−0.0592 (3)	0.48815 (15)	0.0570 (6)	
H32	−0.0373	−0.1189	0.4506	0.068*	
C33	0.01817 (17)	−0.0461 (3)	0.58167 (15)	0.0516 (6)	
H33	−0.0359	−0.0957	0.6067	0.062*	
C34	0.09947 (16)	0.0405 (3)	0.63681 (13)	0.0430 (5)	
H34	0.1007	0.0480	0.6996	0.052*	
O1	0.30602 (9)	0.54559 (13)	0.92705 (7)	0.0281 (3)	
O2	0.44079 (9)	0.71853 (15)	0.82204 (8)	0.0383 (3)	
O4	0.21835 (9)	0.32927 (16)	0.71031 (8)	0.0385 (3)	
O6	0.39918 (10)	0.22905 (16)	0.92224 (9)	0.0376 (3)	
OW	0.55620 (12)	0.4829 (2)	0.94836 (10)	0.0379 (3)	
S1	0.14464 (4)	0.77358 (5)	0.87681 (3)	0.03636 (13)	
HW1	0.528 (2)	0.551 (4)	0.9035 (18)	0.084 (9)*	
HW2	0.5117 (18)	0.417 (3)	0.9508 (13)	0.047 (7)*	
H6	0.4137 (16)	0.142 (3)	0.9571 (13)	0.050 (6)*	

### Atomic displacement parameters (Å^2^)

**Table:**

	*U*^11^	*U*^22^	*U*^33^	*U*^12^	*U*^13^	*U*^23^
C1	0.0308 (10)	0.0249 (9)	0.0353 (10)	0.0021 (8)	0.0090 (8)	0.0008 (7)
C2	0.0287 (10)	0.0296 (10)	0.0436 (10)	0.0051 (8)	0.0111 (8)	0.0099 (8)
C3	0.0279 (10)	0.0449 (11)	0.0295 (9)	0.0067 (9)	0.0089 (8)	0.0063 (8)
C4	0.0231 (10)	0.0371 (11)	0.0284 (9)	0.0003 (8)	0.0037 (7)	−0.0026 (8)
C5	0.0255 (10)	0.0274 (10)	0.0303 (9)	−0.0021 (8)	0.0061 (7)	−0.0032 (8)
C6	0.0313 (10)	0.0333 (10)	0.0333 (8)	−0.0040 (9)	0.0088 (7)	−0.0021 (8)
C8	0.0266 (10)	0.0275 (9)	0.0394 (9)	0.0041 (8)	0.0093 (7)	−0.0005 (9)
C9	0.0277 (11)	0.0346 (11)	0.0477 (11)	0.0026 (8)	0.0048 (8)	0.0003 (8)
C10	0.0356 (11)	0.0380 (12)	0.0662 (14)	0.0049 (9)	0.0232 (10)	0.0098 (10)
C11	0.0597 (14)	0.0457 (12)	0.0468 (11)	0.0112 (12)	0.0238 (10)	0.0057 (11)
C12	0.0497 (13)	0.0427 (12)	0.0427 (11)	0.0021 (10)	0.0073 (9)	−0.0073 (9)
C13	0.0318 (11)	0.0327 (10)	0.0501 (12)	0.0001 (8)	0.0105 (9)	−0.0028 (9)
C14	0.0534 (14)	0.0313 (12)	0.0750 (14)	−0.0061 (10)	0.0314 (11)	−0.0078 (10)
C15	0.0386 (11)	0.0282 (10)	0.0313 (9)	−0.0064 (8)	0.0103 (8)	0.0023 (8)
C16	0.0331 (11)	0.0442 (11)	0.0312 (9)	−0.0001 (9)	0.0024 (8)	0.0017 (8)
C17	0.0458 (13)	0.0565 (14)	0.0441 (11)	0.0070 (11)	0.0165 (10)	0.0045 (10)
C18	0.0344 (13)	0.0552 (15)	0.0942 (17)	0.0017 (12)	0.0198 (12)	0.0163 (14)
C19	0.0390 (14)	0.0482 (14)	0.0751 (16)	−0.0134 (11)	−0.0160 (12)	0.0203 (12)
C20	0.0600 (15)	0.0312 (10)	0.0345 (10)	−0.0123 (10)	−0.0011 (10)	0.0050 (8)
O3	0.0426 (8)	0.0664 (9)	0.0334 (7)	0.0167 (7)	0.0157 (6)	0.0184 (7)
C21	0.031 (2)	0.065 (3)	0.032 (2)	−0.020 (2)	−0.0058 (17)	0.002 (2)
C22	0.036 (3)	0.022 (4)	0.025 (3)	0.011 (3)	0.007 (3)	0.010 (3)
C23	0.049 (5)	0.065 (4)	0.011 (2)	0.006 (4)	0.001 (2)	−0.004 (2)
C24	0.033 (3)	0.046 (3)	0.041 (4)	−0.012 (2)	0.000 (3)	−0.004 (3)
C25	0.052 (4)	0.052 (4)	0.041 (4)	0.009 (3)	0.030 (3)	0.010 (3)
C26	0.080 (5)	0.047 (4)	0.017 (2)	0.012 (4)	0.008 (3)	−0.002 (2)
C27	0.038 (3)	0.036 (3)	0.052 (6)	−0.011 (2)	−0.007 (4)	−0.002 (4)
C21'	0.0536 (15)	0.0354 (13)	0.0370 (14)	0.0045 (11)	0.0144 (11)	0.0001 (11)
C22'	0.0536 (15)	0.0354 (13)	0.0370 (14)	0.0045 (11)	0.0144 (11)	0.0001 (11)
C23'	0.0536 (15)	0.0354 (13)	0.0370 (14)	0.0045 (11)	0.0144 (11)	0.0001 (11)
C24'	0.0536 (15)	0.0354 (13)	0.0370 (14)	0.0045 (11)	0.0144 (11)	0.0001 (11)
C25'	0.0536 (15)	0.0354 (13)	0.0370 (14)	0.0045 (11)	0.0144 (11)	0.0001 (11)
C26'	0.0536 (15)	0.0354 (13)	0.0370 (14)	0.0045 (11)	0.0144 (11)	0.0001 (11)
C27'	0.0536 (15)	0.0354 (13)	0.0370 (14)	0.0045 (11)	0.0144 (11)	0.0001 (11)
C28	0.0364 (11)	0.0497 (13)	0.0494 (11)	0.0013 (10)	0.0092 (9)	−0.0137 (10)
C29	0.0362 (11)	0.0329 (10)	0.0443 (12)	0.0027 (9)	0.0097 (9)	−0.0077 (9)
C30	0.0488 (13)	0.0425 (12)	0.0447 (12)	−0.0036 (10)	0.0152 (10)	−0.0046 (9)
C31	0.0657 (15)	0.0514 (14)	0.0423 (12)	−0.0035 (12)	0.0040 (11)	−0.0069 (10)
C32	0.0486 (15)	0.0532 (15)	0.0629 (14)	−0.0103 (11)	−0.0046 (12)	−0.0147 (11)
C33	0.0439 (14)	0.0417 (13)	0.0709 (15)	−0.0084 (10)	0.0157 (11)	−0.0053 (11)
C34	0.0427 (12)	0.0416 (11)	0.0464 (11)	0.0025 (10)	0.0124 (9)	−0.0043 (9)
O1	0.0309 (7)	0.0244 (6)	0.0286 (6)	−0.0002 (5)	0.0054 (5)	0.0000 (5)
O2	0.0307 (7)	0.0340 (7)	0.0521 (7)	−0.0014 (6)	0.0127 (6)	0.0108 (6)
O4	0.0283 (7)	0.0484 (8)	0.0378 (7)	0.0024 (6)	0.0042 (5)	−0.0151 (6)
O6	0.0350 (8)	0.0285 (8)	0.0494 (7)	0.0019 (6)	0.0082 (6)	0.0079 (6)
OW	0.0299 (8)	0.0340 (8)	0.0469 (8)	−0.0020 (7)	0.0005 (6)	0.0019 (6)
S1	0.0316 (3)	0.0396 (3)	0.0383 (2)	0.0082 (2)	0.00785 (19)	0.0010 (2)

### Geometric parameters (Å, °)

**Table:**

C1—O1	1.414 (2)	O3—C21	1.403 (3)
C1—C2	1.528 (2)	C21—C22	1.496 (6)
C1—S1	1.8355 (17)	C21—H21A	0.9700
C1—H1	0.9800	C21—H21B	0.9700
C2—O2	1.421 (2)	C22—C23	1.366 (8)
C2—C3	1.524 (2)	C22—C27	1.412 (7)
C2—H2	0.9800	C23—C24	1.366 (7)
C3—O3	1.427 (2)	C23—H23	0.9300
C3—C4	1.515 (3)	C24—C25	1.382 (7)
C3—H3	0.9800	C24—H24	0.9300
C4—O4	1.425 (2)	C25—C26	1.377 (8)
C4—C5	1.529 (2)	C25—H25	0.9300
C4—H4	0.9800	C26—C27	1.393 (8)
C5—O1	1.4347 (18)	C26—H26	0.9300
C5—C6	1.506 (2)	C27—H27	0.9300
C5—H5	0.9800	C21'—C22'	1.510 (8)
C6—O6	1.435 (2)	C21'—H21C	0.9700
C6—H6A	0.9700	C21'—H21D	0.9700
C6—H6B	0.9700	C22'—C23'	1.356 (10)
C8—C9	1.381 (2)	C22'—C27'	1.384 (10)
C8—C13	1.386 (2)	C23'—C24'	1.370 (9)
C8—S1	1.7777 (17)	C23'—H23'	0.9300
C9—C10	1.390 (3)	C24'—C25'	1.346 (9)
C9—H9	0.9300	C24'—H24'	0.9300
C10—C11	1.372 (3)	C25'—C26'	1.375 (9)
C10—H10	0.9300	C25'—H25'	0.9300
C11—C12	1.375 (3)	C26'—C27'	1.377 (9)
C11—H11	0.9300	C26'—H26'	0.9300
C12—C13	1.382 (3)	C27'—H27'	0.9300
C12—H12	0.9300	C28—O4	1.403 (2)
C13—H13	0.9300	C28—C29	1.500 (3)
C14—O2	1.417 (2)	C28—H28A	0.9700
C14—C15	1.493 (3)	C28—H28B	0.9700
C14—H14A	0.9700	C29—C30	1.384 (3)
C14—H14B	0.9700	C29—C34	1.389 (3)
C15—C16	1.386 (2)	C30—C31	1.386 (3)
C15—C20	1.388 (3)	C30—H30	0.9300
C16—C17	1.370 (3)	C31—C32	1.365 (3)
C16—H16	0.9300	C31—H31	0.9300
C17—C18	1.368 (3)	C32—C33	1.389 (3)
C17—H17	0.9300	C32—H32	0.9300
C18—C19	1.368 (3)	C33—C34	1.372 (3)
C18—H18	0.9300	C33—H33	0.9300
C19—C20	1.392 (3)	C34—H34	0.9300
C19—H19	0.9300	O6—H6	0.87 (2)
C20—H20	0.9300	OW—HW1	0.88 (3)
O3—C21'	1.362 (4)	OW—HW2	0.78 (2)
			
O1—C1—C2	111.21 (14)	C21—O3—C3	116.2 (2)
O1—C1—S1	114.34 (12)	O3—C21—C22	109.4 (4)
C2—C1—S1	108.15 (11)	O3—C21—H21A	109.8
O1—C1—H1	107.6	C22—C21—H21A	109.8
C2—C1—H1	107.6	O3—C21—H21B	109.8
S1—C1—H1	107.6	C22—C21—H21B	109.8
O2—C2—C3	108.46 (14)	H21A—C21—H21B	108.2
O2—C2—C1	109.45 (13)	C23—C22—C27	119.2 (6)
C3—C2—C1	110.59 (14)	C23—C22—C21	120.9 (5)
O2—C2—H2	109.4	C27—C22—C21	119.5 (5)
C3—C2—H2	109.4	C22—C23—C24	121.0 (5)
C1—C2—H2	109.4	C22—C23—H23	119.5
O3—C3—C4	108.00 (14)	C24—C23—H23	119.5
O3—C3—C2	110.94 (15)	C23—C24—C25	120.4 (4)
C4—C3—C2	111.68 (13)	C23—C24—H24	119.8
O3—C3—H3	108.7	C25—C24—H24	119.8
C4—C3—H3	108.7	C26—C25—C24	120.3 (5)
C2—C3—H3	108.7	C26—C25—H25	119.9
O4—C4—C3	108.98 (12)	C24—C25—H25	119.9
O4—C4—C5	105.68 (14)	C25—C26—C27	119.5 (5)
C3—C4—C5	112.09 (14)	C25—C26—H26	120.2
O4—C4—H4	110.0	C27—C26—H26	120.2
C3—C4—H4	110.0	C26—C27—C22	119.6 (6)
C5—C4—H4	110.0	C26—C27—H27	120.2
O1—C5—C6	106.70 (11)	C22—C27—H27	120.2
O1—C5—C4	111.58 (13)	O3—C21'—C22'	113.2 (5)
C6—C5—C4	112.98 (14)	O3—C21'—H21C	108.9
O1—C5—H5	108.5	C22'—C21'—H21C	108.9
C6—C5—H5	108.5	O3—C21'—H21D	108.9
C4—C5—H5	108.5	C22'—C21'—H21D	108.9
O6—C6—C5	110.21 (14)	H21C—C21'—H21D	107.8
O6—C6—H6A	109.6	C23'—C22'—C27'	117.2 (8)
C5—C6—H6A	109.6	C23'—C22'—C21'	122.1 (10)
O6—C6—H6B	109.6	C27'—C22'—C21'	120.6 (9)
C5—C6—H6B	109.6	C22'—C23'—C24'	122.3 (7)
H6A—C6—H6B	108.1	C22'—C23'—H23'	118.9
C9—C8—C13	119.53 (16)	C24'—C23'—H23'	118.9
C9—C8—S1	117.95 (13)	C25'—C24'—C23'	120.4 (7)
C13—C8—S1	122.40 (14)	C25'—C24'—H24'	119.8
C8—C9—C10	120.02 (17)	C23'—C24'—H24'	119.8
C8—C9—H9	120.0	C24'—C25'—C26'	119.0 (7)
C10—C9—H9	120.0	C24'—C25'—H25'	120.5
C11—C10—C9	120.23 (19)	C26'—C25'—H25'	120.5
C11—C10—H10	119.9	C25'—C26'—C27'	120.3 (8)
C9—C10—H10	119.9	C25'—C26'—H26'	119.9
C10—C11—C12	119.72 (18)	C27'—C26'—H26'	119.9
C10—C11—H11	120.1	C26'—C27'—C22'	120.7 (9)
C12—C11—H11	120.1	C26'—C27'—H27'	119.7
C11—C12—C13	120.66 (17)	C22'—C27'—H27'	119.7
C11—C12—H12	119.7	O4—C28—C29	108.24 (15)
C13—C12—H12	119.7	O4—C28—H28A	110.0
C12—C13—C8	119.79 (18)	C29—C28—H28A	110.0
C12—C13—H13	120.1	O4—C28—H28B	110.0
C8—C13—H13	120.1	C29—C28—H28B	110.0
O2—C14—C15	109.44 (16)	H28A—C28—H28B	108.4
O2—C14—H14A	109.8	C30—C29—C34	118.92 (17)
C15—C14—H14A	109.8	C30—C29—C28	120.18 (18)
O2—C14—H14B	109.8	C34—C29—C28	120.90 (17)
C15—C14—H14B	109.8	C29—C30—C31	120.1 (2)
H14A—C14—H14B	108.2	C29—C30—H30	119.9
C16—C15—C20	118.96 (19)	C31—C30—H30	119.9
C16—C15—C14	120.41 (16)	C32—C31—C30	120.32 (19)
C20—C15—C14	120.61 (18)	C32—C31—H31	119.8
C17—C16—C15	120.65 (18)	C30—C31—H31	119.8
C17—C16—H16	119.7	C31—C32—C33	120.20 (19)
C15—C16—H16	119.7	C31—C32—H32	119.9
C18—C17—C16	120.4 (2)	C33—C32—H32	119.9
C18—C17—H17	119.8	C34—C33—C32	119.5 (2)
C16—C17—H17	119.8	C34—C33—H33	120.2
C19—C18—C17	119.9 (2)	C32—C33—H33	120.2
C19—C18—H18	120.0	C33—C34—C29	120.89 (18)
C17—C18—H18	120.0	C33—C34—H34	119.6
C18—C19—C20	120.51 (19)	C29—C34—H34	119.6
C18—C19—H19	119.7	C1—O1—C5	114.61 (11)
C20—C19—H19	119.7	C14—O2—C2	116.02 (14)
C15—C20—C19	119.52 (19)	C28—O4—C4	116.34 (13)
C15—C20—H20	120.2	C6—O6—H6	106.8 (14)
C19—C20—H20	120.2	HW1—OW—HW2	106 (2)
C21'—O3—C21	40.1 (2)	C8—S1—C1	101.91 (8)
C21'—O3—C3	115.02 (19)		

### Hydrogen-bond geometry (Å, °)

**Table:**

*D*—H···*A*	*D*—H	H···*A*	*D*···*A*	*D*—H···*A*
OW—HW1···O2	0.88 (3)	2.00 (3)	2.861 (2)	166 (2)
OW—HW2···O6	0.78 (2)	2.07 (2)	2.827 (2)	165 (2)
O6—H6···OW^i^	0.87 (2)	1.89 (2)	2.745 (2)	169 (2)

Symmetry codes: (i) −*x*+1, *y*−1/2, −*z*+2.
